# What Renders TAU Toxic

**DOI:** 10.3389/fneur.2013.00072

**Published:** 2013-06-10

**Authors:** Jürgen Götz, Di Xia, Gerhard Leinenga, Yee Lian Chew, Hannah R. Nicholas

**Affiliations:** ^1^Centre for Ageing Dementia Research, Queensland Brain Institute, The University of Queensland, Brisbane, QLD, Australia; ^2^Sydney Medical School, Brain and Mind Research Institute, University of Sydney, Sydney, NSW, Australia; ^3^School of Molecular Bioscience, University of Sydney, Sydney, NSW, Australia

**Keywords:** Alzheimer’s disease, *C. elegans*, frontotemporal dementia, knock-out, PP2A, PTL-1, TAU, transgenic

## Abstract

TAU is a microtubule-associated protein that under pathological conditions such as Alzheimer’s disease (AD) forms insoluble, filamentous aggregates. When 20 years after TAU’s discovery the first TAU transgenic mouse models were established, one declared goal that was achieved was the modeling of authentic TAU aggregate formation in the form of neurofibrillary tangles. However, as we review here, it has become increasingly clear that TAU causes damage much before these filamentous aggregates develop. In fact, because TAU is a scaffolding protein, increased levels and an altered subcellular localization (due to an increased insolubility and impaired clearance) result in the interaction of TAU with cellular proteins with which it would otherwise either not interact or do so to a lesser degree, thereby impairing their physiological functions. We specifically discuss the non-axonal localization of TAU, the role phosphorylation has in TAU toxicity and how TAU impairs mitochondrial functions. A major emphasis is on what we have learned from the four available TAU knock-out models in mice, and the knock-out of the TAU/MAP2 homolog PTL-1 in worms. It has been proposed that in human pathological conditions such as AD, a rare toxic TAU species exists which needs to be specifically removed to abrogate TAU’s toxicity and restore neuronal functions. However, what is toxic in one context may not be in another, and simply reducing, but not fully abolishing TAU levels may be sufficient to abrogate TAU toxicity.

## Introduction

TAU belongs to the family of microtubule-associated proteins (MAPs) (Dehmelt and Halpain, 2005). MAPs act in concert with heterodimers of α- and β-tubulin to assemble microtubules. They were named according to the three major size classes of polypeptides: MAP1 (>250 kDa), MAP2 (∼200 kDa), and TAU (50–70 kDa) (Dehmelt and Halpain, 2005; Halpain and Dehmelt, 2006). MAP2 and TAU are expressed together in most neurons, where they localize to separate subcellular compartments. MAP2 is largely found in dendrites, whereas TAU is concentrated in axons (Matus, 1990). TAU has also been found in astrocytes and oligodendrocytes, although, under physiological conditions, its levels are relatively low (Tashiro et al., 1997). In concert with actin and intermediate filaments, microtubules establish and maintain the overall internal architecture of the cytoplasm and thereby comprise a major determinant of overall cell shape (Allen et al., 1985; Vale et al., 1985; Nangaku et al., 1994; Trinczek et al., 1999). Besides interacting with cytoskeletal proteins, MAPs also interact with proteins that have a range of cellular functions, suggesting that TAU is a scaffolding protein (Brandt and Leschik, 2004). Scaffolding proteins are defined as being able to bind at least two signaling proteins, and localizing signaling molecules and transduction pathways to defined cellular locations – for instance the cell body, dendrites or the axon, and to regulate signaling (Shaw and Filbert, 2009). We discuss here that TAU has these properties and that the toxicity of TAU is, at least in parts, because this scaffolding function is either altered or lost.

When TAU was discovered in 1975 (Weingarten et al., 1975), the subsequent years focused mainly on its tissue distribution and role in microtubule assembly and stabilization. The focus shifted radically when TAU was identified in Alzheimer’s disease (AD) brains in a highly phosphorylated form as the filamentous core of the neurofibrillary tangles (NFTs) (Grundke-Iqbal et al., 1986; Goedert et al., 1988). Histopathologically, AD is also characterized by β-amyloid (Aβ)-containing plaques, reduced synaptic density, and neuronal loss in selected brain areas. Interestingly, although NFTs were initially correlated with dementia (Arriagada et al., 1992), it was also found that firstly, CA1 hippocampal neurons can survive with NFTs for about 20 years, and secondly that NFTs may not be obligatory for the death of CA1 hippocampal neurons in AD (Morsch et al., 1999). This does not, however, preclude that NFT-carrying neurons are functionally impaired. For a discussion of the role of NFTs in neurodegeneration, see e.g., Castillo-Carranza et al. (2013). In addition to AD, neurofibrillary lesions are also abundant in other neurodegenerative diseases such as Pick’s disease, progressive supranuclear palsy (PSP), corticobasal degeneration (CBD), argyrophilic grain disease (AGD), and frontotemporal dementia with Parkinsonism linked to chromosome 17 (FTDP-17), where they occur in the absence of overt Aβ deposition. When we, for the first time, expressed human TAU in transgenic mice, we were able to reproduce key aspects of the human TAU pathology (Götz et al., 1995). Specifically, overexpression of human TAU resulted in the accumulation of hyperphosphorylated forms of TAU not only in the axonal compartment, but also in the somatodendritic domain. NFT formation, however, was not achieved by this approach. It was the identification of pathogenic mutations in familial cases of frontotemporal lobar degeneration (FTLD) in the *MAPT* gene encoding TAU (Clark et al., 1998; Hutton et al., 1998; Poorkaj et al., 1998) that enabled us and others to express FTLD mutant forms of TAU in mice. Thus, we not only reproduced NFT formation, but in addition placed TAU downstream of Aβ in a patho-cascade – a central concept in the field (Götz et al., 2001a,b). Despite this, TAU is not simply an innocent bystander in Aβ toxicity, as discussed below. In fact, the dendritic localization of the “axonal” protein TAU not only has a role in physiological postsynaptic scaffolding, but it is also crucial for mediating Aβ’s toxicity (Ittner et al., 2010). This and complementary studies in cell culture have put forth the argument that reducing or completely abolishing TAU expression might be beneficial in combating neurodegeneration in AD (Rapoport et al., 2002; Roberson et al., 2007; Ittner and Götz, 2011; Morris et al., 2011b).

In the following, we discuss what we have learned about TAU’s toxicity in models that over-express TAU and as a consequence, develop TAU aggregates and NFTs. As this article is part of a series that investigates TAU toxicity from various angles, we will restrict our discussion to selected aspects and will specifically discuss TAU’s role in non-axonal compartments, how it affects mitochondrial functions and what we have learned from invertebrate models. We draw the conclusion that TAU is a scaffolding protein and that its mislocalization disrupts cellular processes because it traps proteins with which it would otherwise not interact (or do so to a lesser degree) and thereby prevents them from executing their physiological functions. This does not rule out that the aggregates themselves are toxic, although the current experimental models do not allow for a dissociation of the effects elicited by the aggregates from those elicited by soluble TAU species. In the second part we discuss what we have learned from TAU knock-out models in mice and what the knockout of the worm homolog of TAU, PTL-1, has contributed to an understanding of TAU function. In the final paragraph, we attempt to integrate findings in both TAU over-expressing and knock-out models.

## What Causes Toxicity When There is Too Much TAU?

In an attempt to model TAU pathology in mice, the first TAU transgenic mouse model was established 20 years after TAU’s discovery by over-expressing the longest of the six major human TAU isoforms, hTau40 (Götz et al., 1995) (For comparison, in the adult mouse brain, there are only three TAU isoforms expressed.). These htau40 transgenic mice reproduce aspects of the human pathology, such as somatodendritic localization and hyperphosphorylation of TAU. Subsequently, stronger promoters were used to drive expression, resulting in more pronounced phenotypes (Ishihara et al., 1999; Spittaels et al., 1999; Probst et al., 2000). Despite the formation of TAU aggregates and an age-dependent decrease in TAU solubility, NFTs did not form until a very old age in these mice (Ishihara et al., 2001). However, signs of Wallerian degeneration including axonal breakdown and segmentation of myelin into ellipsoids were observed. Furthermore, neurogenic muscle atrophy, with groups of small angular muscle fibers, was present in the hind leg musculature of the transgenic mice (Probst et al., 2000). Another characteristic was the presence of large numbers of axonal spheroids in brainstem and spinal cord (Ishihara et al., 1999; Spittaels et al., 1999; Probst et al., 2000). Similar swellings have also been described in transgenic mice with Aβ plaque formation, and in the human AD brain (Stokin et al., 2005).

Following the identification of pathogenic mutations in *MAPT* in FTDP-17, TAU filament formation was achieved in neurons and glia, both by constitutive and doxycycline-regulated expression of mutant human forms of TAU (Götz and Götz, 2009). The first published mouse strain with pronounced NFT formation, JNPL3, expressed P301L mutant human TAU under the control of the murine PrP promoter. This resulted in abnormal TAU filament formation in neurons as well as astrocytes and oligodendrocytes, with NFTs being present in brain and spinal cord (Lewis et al., 2000). Neuronal loss was found in the spinal cord, as evidenced by a twofold reduction in the numbers of motor neurons. Furthermore, the mice developed severe motor disturbances by 10 months of age. We established a second model with NFT formation, pR5 mice, by expressing the same P301L mutation, but using a different human TAU isoform and the neuron-specific mThy1.2 promoter for transgene expression (Götz et al., 2001a). While a motor phenotype was absent, reference memory was impaired and the mice displayed an increased exploratory behavior (Pennanen et al., 2006). Furthermore, owing to a pronounced expression of the transgene in the amygdala, the mice showed an accelerated extinction in the conditioned taste aversion paradigm (Pennanen et al., 2004). When experimental diabetes was induced in pR5 mice, this caused an earlier-onset and increased formation of NFTs, indicating that diabetes can accelerate the onset and increase the severity of disease in individuals with a predisposition to develop a tauopathy (Ke et al., 2009). To determine whether NFTs are integral components of the neurotoxic cascade in AD or whether they represent a protective neuronal response, transgenic mice were generated which allowed for the regulation of P301L TAU expression by adding doxycycline to the drinking water (Santacruz et al., 2005). Because the system resulted in a 15-fold over-expression, this caused a progressive formation of NFTs, a remarkable neuronal loss (70% in the CA1 region), gross atrophy of the brain, and behavioral impairment. Turning the system “off” caused a reduction of TAU levels to 2.5-fold over-expression. Nonetheless, this was sufficient to cause a recovery of memory functions and stabilization of neuron numbers, while NFTs continued to accumulate. These data imply that NFTs *per se* are not sufficient to cause cognitive decline or neuronal death (Santacruz et al., 2005).

While the pR5 mice display memory impairment as a major clinical feature of AD, another feature, Parkinsonism, that characterizes a significant subset of FTLD cases, has been modeled in K369I mutant TAU transgenic K3 mice. We established this strain based on the identification of the K369I mutation of TAU in a single patient with Pick’s disease (Neumann et al., 2001), and reproduced the distinct characteristics of Pick’s pathology in mice (Ittner et al., 2008). Memory functions were impaired as shown in the novel object recognition test. Owing to a unique expression pattern of the transgene that extends to the Substantia Nigra pars compacta (SNpc), the K3 mice also model early onset Parkinsonism, i.e., resting tremor, bradykinesia, postural instability, and gait anomalies. They show an increased cataleptic response to haloperidol and an early, but not late, response to l-DOPA, indicating that the dopaminergic system is impaired. We found a selectively impaired axonal transport of distinct cargos including mitochondria and tyrosine hydroxylase (TH)-containing vesicles. An important finding is that at the molecular level, this transport impairment is the result of the accumulation of hyperphosphorylated TAU in the cell body, where it traps the adapter protein and a component of the kinesin motor machinery, JIP1, thereby preventing this molecule from executing its physiological function in the axon (Ittner et al., 2008). A pathological interaction between TAU and JIP1 was further revealed in AD and not control brain (Ittner et al., 2009). It seems therefore that for TAU to be able to exert toxicity, the subcellular compartments it is aberrantly targeted to are crucial, as this determines with which proteins it pathologically interacts and which cellular functions it impairs. In this process, phosphorylation of TAU has a critical role as reviewed by us recently (Götz et al., 2010). That phosphorylation of TAU is critical in toxicity is also evident from studies in protein phosphatase 2A (PP2A) dominant negative mutant strains. More specifically, to address the role of the TAU phosphatase PP2A in mice with a pre-existing TAU pathology, we crossed the PP2A dominant negative mutant strain Dom5 with pR5 mice that express P301L mutant TAU and found that this exacerbated the TAU pathology of pR5 mice significantly. The double-transgenic mice showed sevenfold increased numbers of hippocampal neurons that specifically phosphorylated the pathological Ser422 epitope of TAU (Deters et al., 2009). The mice showed eightfold increased numbers of NFTs compared with pR5 mice, in agreement with our previous finding that NFT formation is correlated with and preceded by phosphorylation of TAU at the Ser422 epitope (Götz et al., 2001b). We further used the Dom5 mice to show that a small compound, sodium selenate, improves TAU-dependent impairment and neurodegeneration in a PP2A-dependent manner (van Eersel et al., 2010). Together this demonstrates that phosphorylated forms of TAU have a critical role in TAU toxicity.

The tauopathies PSP and CBD are characterized by substantial glial TAU pathology. Aspects of this pathology have been reproduced by expressing G272V mutant TAU under the control of the PrP promoter that resulted in high transgene expression in a subset of both neurons and oligodendrocytes. Electron microscopy established that TAU filament formation was associated with hyperphosphorylation of TAU. Thioflavin S-positive fibrillary inclusions were identified in oligodendrocytes and motor neurons (Götz et al., 2001c), the clinical phenotype of these mice however was subtle. In contrast, when human wild-type TAU was overexpressed in neurons and glial cells under the control of the mouse Tα1 α-tubulin promoter, a glial pathology was obtained that closely resembled the astrocytic plaques of CBD and the coiled bodies of both CBD and PSP (Higuchi et al., 2002). A significant age-related neuronal loss was only found at 18 months of age, whereas oligodendrocytes were lost already at 6 months. The same team employed the 2′,3′-cyclic nucleotide 3′-phosphodiesterase promoter to express human P301L TAU exclusively in oligodendrocytes (Higuchi et al., 2005). Interestingly, the structural disruption of myelin and axons preceded the emergence of TAU inclusions in oligodendrocytes; also, impaired axonal transport was found to precede the motor deficits in these mice (Higuchi et al., 2005). Together, these studies highlight a role for glial TAU in disease.

While most studies on TAU use the mouse as a model organism, several wild-type and mutant human TAU transgenic models have been established in the nematode *C. elegans* (Kraemer et al., 2003; Miyasaka et al., 2005; Brandt et al., 2009; Fatouros et al., 2012). Some of these models have been reviewed in detail (Ewald and Li, 2010) and are not discussed here.

In the following, we will discuss two important aspects of TAU toxicity, one is the impairment of mitochondrial functions, and the second the role TAU has in the dendrite. Mitochondrial dysfunction has long been associated with the pathophysiology of AD (Blass and Gibson, 1991). The morphology of a cellular mitochondrial network is maintained by reciprocal rounds of fission and fusion, a process termed mitochondrial dynamics. Elevated fusion produces elongated, interconnected mitochondria, while enhanced fission results in mitochondrial fragmentation. Mitochondrial dynamics cannot be discussed in isolation, as fission (biogenesis), fusion, bioenergetics, motility/transport, and turnover by mitophagy are highly inter-dependent processes (Chen and Chan, 2009). How TAU impairs the fission and fusion of mitochondria, has been reviewed by us recently (Duboff et al., 2013), following the finding of a role of TAU in altering mitochondrial dynamics. Specifically we had found that in human TAU transgenic mice and flies, F-actin is increased, which disrupts the physical association of mitochondria and the fission protein DRP1, leading to mitochondrial elongation (Duboff et al., 2012). The resulting neurotoxicity can be rescued either by reducing mitochondrial fusion, or by enhancing fission, or by reversing actin stabilization.

Earlier, we had analyzed P301L TAU transgenic pR5 mice by MALDI TOF/TOF mass-spectrometric analysis, which revealed a deregulation of mainly metabolism-related proteins including mitochondrial respiratory chain complex components such as the complex V component ATP synthase D chain, antioxidant enzymes, and synaptic proteins (David et al., 2005). A subsequent functional analysis demonstrated a mitochondrial dysfunction in pR5 mice together with a reduced NADH-ubiquinone oxidoreductase activity and, with age, impaired mitochondrial respiration and ATP synthesis. Mitochondrial dysfunction was associated with higher levels of reactive oxygen species (ROS) in aged transgenic mice and an up-regulation of antioxidant enzymes. When complex V levels were analyzed in human FTDP-17 patient brains carrying the P301L TAU mutation, a significant decrease in complex V levels was found in all P301L human brain samples compared with controls, underscoring the validity of the proteomics findings in mice for the human disease (David et al., 2005). A serial analysis of gene expression (SAGE) of the same mouse strain looking specifically at the amygdala also revealed deregulated mitochondrial genes (Ke et al., 2012a).

Mitochondrial functions are also impaired by Aβ and as there are many findings that support the notion of a cross-talk of Aβ and TAU in affecting mitochondrial functions (Eckert et al., 2010), it was reasonable to cross pR5 mice with Aβ plaque-forming APP^sw^PS2^N141I^ double-transgenic APP152 mice to generate triple-transgenic (^triple^AD) mice that combine both pathologies in one model (Grueninger et al., 2010). Quantitative isobaric-tag labeling (iTRAQ) followed by mass spectrometry revealed a massive deregulation of 24 proteins, of which one third were mitochondrial proteins mainly related to complexes I and IV of the oxidative phosphorylation system (OXPHOS). When mitochondrial functions were addressed, this revealed that deregulation of complex I is TAU-dependent, whereas deregulation of complex IV is Aβ-dependent, both at the protein and activity level (Rhein et al., 2009). In addition, synergistic effects of Aβ and TAU were evident in the ^triple^AD mice establishing a molecular link between Aβ and TAU protein in AD pathology *in vivo* (Rhein et al., 2009). In the P301L TAU transgenic pR5 mice, a transcriptomic analysis further revealed an up-regulation of glyoxalase I that detoxifies dicarbonyl compounds and thereby reduces the formation of advanced glycation end (AGE) products (Chen et al., 2004). Co-staining with a phospho-TAU antibody suggested glyoxalase I up-regulation as an early defense mechanism to combat elevated levels of aggregated TAU. To understand which processes are disrupted by Aβ in the presence of TAU aggregates, we applied comparative proteomics to Aβ-treated P301L TAU-expressing neuroblastoma cells and the amygdala of P301L TAU transgenic pR5 mice that had been stereotaxically injected with Aβ preparations. We found that a significant fraction of proteins that were altered in both systems belonged to the same functional categories, i.e., proteins involved in the stress-response associated with protein folding (David et al., 2006). Among the deregulated proteins was valosin containing protein (VCP), an essential component of the ER-associated degradation (ERAD) process, whereas members of the peroxiredoxin family were down-regulated. Together this indicates that TAU and Aβ exert both separate and synergistic toxic effects that are mediated by mitochondria and the stress-related unfolded protein response.

The amyloid cascade hypothesis in a patho-cascade places Aβ upstream of TAU (Hardy, 2006). This concept has been proven in P301L mutant TAU transgenic mice that develop an increased number of NFTs, either by crossing them with Aβ plaque-forming transgenic mice (Lewis et al., 2001), or by stereotaxically injecting Aβ into their brains (Götz et al., 2001b). That Aβ toxicity is dependent on TAU has first been shown *in vitro* because primary neuronal cultures derived from TAU knock-out mice were resistant to the toxic effects of Aβ (Rapoport et al., 2002). This finding was subsequently reproduced *in vivo*, by crossing Aβ plaque-forming mice that are characterized by premature mortality, high susceptibility to experimentally induced excitotoxic seizures and memory deficits, onto a TAU knock-out background (Roberson et al., 2007). Mechanistically, this protection appeared to be conferred by a reduced susceptibility to excitotoxicity when TAU was either absent or when its levels were reduced (Roberson et al., 2007). Excitotoxicity is the over-activation of NMDA receptors (NMDARs) that results in neuronal damage and death because of increased calcium influx and nitric oxide (NO) activation (Palop and Mucke, 2009). By using another APP plaque-forming mouse strain and a different TAU knock-out strain, we were able to reproduce the TAU-dependent protection from Aβ-induced premature mortality and memory deficits, and determined that this protection is conferred by a reduced susceptibility to excitotoxicity (Ittner et al., 2010). Likewise, overexpression of a truncated form of TAU (ΔTau) that lacks the microtubule-binding region MBR also rescued the phenotype of the Aβ plaque-forming mice (Ittner et al., 2010). In a more recent model, tau pathology was shown to develop independent of Aβ (Winton et al., 2011). This was shown by finding no differences in TAU lesion formation when crossing 3xTg-AD mice that co-express mutant forms of APP, presenilin and TAU and hence develop a plaque and TAU pathology, with a mouse strain that lacks the enzyme BACE1 required for Aβ generation. However, it has to be considered that the TAU pathology in the 3xTg-AD mice develops very late and is modest compared with that of other widely used TAU transgenic mouse models (Oddo et al., 2003a,b).

What is the mechanistic explanation for this protection conferred by reduction of TAU? We next showed that TAU is also present in the dendrite (although in low quantities compared with the axon), where it is critically involved in postsynaptic NMDAR downstream signaling by localizing the SRC kinase FYN to the dendrite. FYN phosphorylates the NMDAR that then recruits the postsynaptic scaffolding protein PSD-95 to form a complex. Because levels of postsynaptic FYN are massively reduced in TAU-deficient or ΔTau-expressing mice, this results in uncoupling of NMDARs from excitotoxic signaling and abrogation of Aβ-mediated toxicity (Ittner et al., 2010; Ittner and Götz, 2011). A subsequent study found that Aβ causes a range of deleterious effects on TAU including its localization to dendrites (Zempel et al., 2010). Aβ, Fyn, and TAU therefore seem to orchestrate neuronal damage (Haass and Mandelkow, 2010; Ittner et al., 2010; Roberson et al., 2011). An essential role for FYN in this process is supported by the fact that the pathology of Aβ plaque-forming mice is enhanced when FYN is overexpressed and ameliorated when FYN is absent (Chin et al., 2004, 2005).

TAU interacts with FYN at least in two ways. Firstly, although TAU is mainly phosphorylated on serine and threonine residues, it also contains three tyrosine residues. Tyr18 is phosphorylated by FYN, and the phosphorylated motif interacts with FYN via FYN’s SH2 domain. Secondly, TAU contains seven PXXP motifs located in the amino-terminus (all of which are retained in the ΔTau construct). Of these, the seventh proline-rich RTPPKSP motif has been shown to be crucial for the interaction with the SH3 domain of FYN and other SRC non-receptor tyrosine kinases (Lee et al., 1998; Bhaskar et al., 2010; Ittner et al., 2010). Interestingly, this motif is also critical in the interaction of TAU with the phosphatase PP2A that exists as a heterotrimeric holoenzyme complex. It has been shown recently that the PP2A regulatory subunit Bα binds to and dephosphorylates TAU, and thereby regulates microtubule stability (Sontag et al., 2012). When FYN is bound to the (seventh) proline-rich RTPPKSP motif that is conserved in both TAU and MAP2, this inhibits the interaction of PP2A/Bα with either TAU or MAP2. The corresponding synthetic RTPPKSP peptide, but not the phosphorylated RpTPPKSP version, competes with TAU and MAP2 for binding to PP2A/Bα. This finding is remarkable because the down-regulation of PP2A/Bα and the deregulation of FYN/TAU interactions have been linked to enhanced TAU phosphorylation in AD (Sontag et al., 2012). MAP2 is mainly a dendritic protein as mentioned above. Why MAP2 cannot compensate for the absence of TAU in TAU knock-out mice and target FYN to the dendritic spine is not understood, especially as both MAP2 and TAU have been shown to efficiently bind to FYN.

## What are the Effects of Reduced TAU Levels?

As reviewed in detail recently, a series of TAU knock-out mouse strains have been generated to gain insight into the physiological functions of TAU, and while initially reported to be overtly normal, behavioral changes and motor deficits were identified at least for some of these strains (Ke et al., 2012b).

Work in primary neuronal cultures laid the foundation: when TAU was down-regulated using antisense oligonucleotides, this caused a reduced neuronal outgrowth (Caceres and Kosik, 1990). However, the first TAU knock-out mice established by Harada et al. (1994) a few years later were surprisingly normal, with no evidence of either an altered axonal elongation or any macroscopic change. In a compensatory mechanism for the absence of TAU, MAP1A was up-regulated, and the axon caliber was altered to what is typically found in dendrites. Primary neuronal cultures established from these TAU knock-out mice did not display a reduced neuronal outgrowth phenotype, contrasting with earlier observations of neurons in which TAU levels had been reduced using antisense approaches (Harada et al., 1994; Takei et al., 2000). Nonetheless, the absence of TAU is not without consequences as the hypoplasticity of the commissural tract and the disorganization of the neuronal layering found in MAP1B knock-out mice (Takei et al., 1997) is exacerbated by cross-breeding these mice with TAU knock-out mice (Takei et al., 2000).

In 2001, two additional TAU knock-out lines became available. The first was established by Dawson et al. (2001), who used the same strategy as Harada and colleagues by replacing the first coding exon of TAU with a neomycin selection cassette, thereby abrogating TAU expression. The second “knock-in” strain was generated by Tucker et al. (2001), who inserted a GFP cassette in frame, resulting in a fusion protein that contained the first 31 amino acids of TAU. None of these two strains revealed any obvious impairment [at the time, the Tucker strain was only used as a “tool” to study neurotrophins; we have, however, since used this strain for behavioral studies (Ittner et al., 2010)]. Interestingly, in the Dawson strain, MAP1A levels were increased twofold at birth, while they were reduced back to normal levels as the mice became older suggesting that MAP1A may compensate for the loss of TAU during early brain development, but not in the mature brain (Dawson et al., 2001). In keeping with the very first *in vitro* studies (Caceres and Kosik, 1990), primary neurons obtained from this knock-out strain showed a slowed maturation with reduced neurite length throughout all developmental stages and a reduced axon length of stage 3 (onset of polarity) neurons (Dawson et al., 2001). A fourth TAU knockout was established in 2007, again by inserting a selection cassette into exon 1, but the cassette was flanked by FRT (flippase recognition target) recombination motifs to allow for subsequent manipulation of the targeted *MAPT* gene (Fujio et al., 2007). Not surprisingly, the mice looked quite normal, and MAP1A levels were found to be increased as previously reported in two of the three knock-out strains. Taken together, TAU is not essential in mice, although small differences were found between the four strains with regards to compensatory mechanisms and when analyzed *ex vivo*.

How do TAU knock-out mice fare in behavioral and motor studies? All four strains presented with no overt phenotype up to 8 months of age (Harada et al., 1994; Dawson et al., 2001; Fujio et al., 2007; Roberson et al., 2007; Ittner et al., 2010; Lei et al., 2012). At 10–12 months, the Dawson mice performed like wild-type in the radial arm and the Morris water maze (Dawson et al., 2010). Subtle motor deficits were detected at 3–3.5 months when the knock-out mice showed an increased latency to cross a beam and also made more slipped steps, but otherwise showed normal motor functions (Morris et al., 2011a). However, at 12 months of age, the Harada mice displayed signs of muscle weakness in the wire-hanging test, reduced balance in the rod-walking test, hyperactivity in a novel environment and impaired contextual fear conditioning (Ikegami et al., 2000). Interestingly, muscle weakness was also evident in heterozygous knock-out mice. Spatial learning, however, of this TAU knock-out strain was normal, when assessed in the eight-arm radial and the Morris water maze (Ikegami et al., 2000). The most pronounced phenotype was recently reported in the Dawson strain kept on a C57BL/6/SV129 background, with Parkinsonism evident at 12 months of age due to a massive loss of dopaminergic neurons in the SNpc, associated with motor impairments such as a reduced performance on the Rotarod and decreased locomotion in the open field (Lei et al., 2012). Because these findings contradict the earlier studies, Morris et al. (2013) performed a detailed behavioral and motor analysis of the Dawson strain that was kept on a C57BL/6 background. They found in their mice that the chronic lack of TAU did not impair learning and memory functions in the Morris water maze and the novel object recognition test, neither at 11–17 nor at 21–22 months of age (Morris et al., 2013). Interestingly, at 12–15 months the knock-out mice weighed more (with 21–22 month-old mice showing a trend) (Morris et al., 2013), while K369I TAU over-expressing mice weigh less (Ittner et al., 2008). The knockout did not alter rearing or activity in the open field, however, both aging and TAU ablation reduced the latency to fall off the Rotarod and this was correlated with body weight. At 12–15 months, TAU knock-out mice took longer to descend from the pole than heterozygous knockout or wild-type mice, whereas for the older age group latencies did not differ. Together this suggests that complete TAU ablation causes subtle motor deficits that are related to an increased body weight. Finally, the newer study did find up to 16% reductions in dopamine levels but this was not sufficient to cause Parkinsonism nor did the administration of l-DOPA improve the performance of the mice in the pole test (Morris et al., 2013). In contrast to the work by Lei et al. (2012), no iron accumulation was found in brain areas such as the hippocampus, striatum or SN. A reason for these discrepancies may be the difference in the genetic background (C57BL/6/SV129 versus C57BL/6) as this has been reported to affect brain metal levels in both transgenic and normal mice (Maynard et al., 2006).

It is apparent that differences in genetic background and design of the targeting construct have an impact on the phenotype of transgenic animals. Another confounding factor for the study of TAU is the presence of other MAPs, which appear to share several biological roles (Dehmelt and Halpain, 2005; Sontag et al., 2012). Potential compensatory functions attributable to closely related MAPs can be excluded in the nematode *C. elegans*, where protein with TAU-like repeats (PTL-1) is the sole homolog of TAU/MAP2/MAP4 in the worm (McDermott et al., 1996; Gordon et al., 2008), meaning that shared physiological functions of the different family members can be addressed.

**Figure 1:**
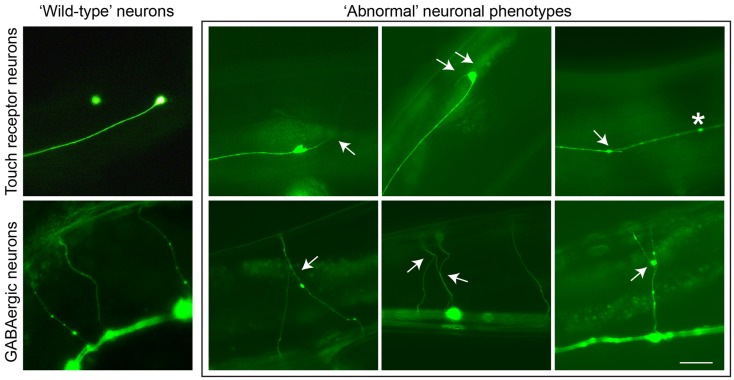
**Neuronal aging in C. elegans is demonstrated by the accumulation of abnormal neuronal structures**. Reporter lines expressing GFP in particular neuronal subsets are used to track these phenotypes as animals age. In touch receptor neurons (top), branching from the cell body or axon, as well as beading along the axon, can be observed. In GABAergic motor neurons (bottom), branching from commissures extending dorsally can be visualized. These phenotypes usually accumulate in late adulthood in wild-type animals, but in *ptl-1* mutant strains these structures can be seen starting in early to mid-adulthood. Arrows indicate branching, asterisks indicate beading. Scale bar = 50 μ. Ventral is down.

PTL-1 contains a high level of sequence homology to TAU/MAP2/MAP4 within the microtubule-binding repeat (MBR) domain in the carboxy-terminus. Analysis of a *ptl-1* transcriptional reporter line demonstrates that PTL-1 has a neuronal expression pattern in adult worms, and in addition PTL-1 has been shown to regulate microtubule assembly *in vitro* (Goedert et al., 1996; McDermott et al., 1996). PTL-1 has been implicated in the regulation of microtubule-based motility in several neurons (Tien et al., 2011) and in the response to gentle touch (Gordon et al., 2008). Aging-related damage in *C. elegans* neurons is evident by neurons displaying abnormal structures such as branching or blebbing from the cell body or axon (Figure 1), and these changes progressively accumulate as the worms age (Pan et al., 2011; Tank et al., 2011; Toth et al., 2012). Using two *ptl-1* mutant strains, one a null knockout (Gordon et al., 2008) and the other putatively generating a protein product containing only the N-terminal region [i.e., similar to our ΔTau truncation construct (Ittner et al., 2010)], it was observed that *ptl-1* mutant strains showed an increased incidence of abnormal structures compared with wild-type animals (Chew et al., 2013). This suggests that the neurons of *ptl-1* mutant animals develop signs of aging at a faster rate than in wild-type animals. In addition, *ptl-1* mutant animals are also short-lived compared with wild-type controls (Chew et al., 2013). These phenotypes of accelerated neuronal aging and shortened organismal lifespan were rescued by re-expressing PTL-1 in a *ptl-1* null mutant. Interestingly, increasing the number of gene copies of *ptl-1* by incorporating the *ptl-1* transgene in a wild-type background was observed to mirror both the neuronal and lifespan phenotypes observed in the *ptl-1* mutant strains, indicating that gene dosage of PTL-1 is vital. This demonstrates a key role of PTL-1 in maintaining neuronal health with age and in regulating whole organism lifespan (Chew et al., 2013). These findings in *C. elegans* show that levels of PTL-1 need to be tightly regulated, suggesting that therapeutic strategies involving the reduction of TAU levels should not lead to a complete reduction of TAU.

## Concluding Remarks

What causes sporadic forms of AD is not known, but it is tempting to speculate that Aβ and TAU act through a combination of excitotoxicity, inhibition of axonal transport and aberrant localization as well as combined effects on mitochondria. It is at the synapse where Aβ induces damage and impairs memory-related electrophysiological properties (Masliah, 1995; Arendt, 2009; Wu et al., 2010). Under basal conditions, mild activation of the NMDAR results in physiological ROS production, while under neurodegenerative conditions, triggered by Aβ, over-activation of NMDARs causes excessive calcium influx and generates NO (Nakamura and Lipton, 2011). These changes in calcium can affect mitochondria (Stanika et al., 2012). Furthermore, axonal transport of cargoes including mitochondria is impaired when TAU detaches from the microtubules and localizes to the somatodendritic domain (Ishihara et al., 1999; Ittner et al., 2008). It has been proposed that in disease conditions such as AD, rare species of toxic TAU exist that need to be removed in order to restore neuronal functions. Other possibilities include a role for non-coding RNAs including mi-RNAs and truncated forms of TAU in toxicity, as discussed elsewhere (Schonrock et al., 2010; Hebert et al., 2012; Zilka et al., 2012). As discussed here, simply reducing TAU levels may be therapeutically beneficial. Work in TAU knock-out mice and PTL-1 knock-out worms suggests that especially in an aging brain, one would not aim for a full ablation of TAU expression. This is also because the TAU/FYN interaction is required for oligodendrocyte functions including myelination. Expression of the N-terminal domain of TAU (a construct similar to ΔTau) alone causes abnormal sorting of FYN, poor myelination and seizures (Klein et al., 2002). However, if one were to succeed in reducing TAU levels by any form of therapeutic intervention, in practical terms it is unlikely that this reduction will be complete. For a positive therapeutic outcome, it might even be sufficient to reduce TAU expression after a pathology has developed as suggested by studies in mice with inducible TAU expression, including those that express shorter variants of TAU (Van der Jeugd et al., 2012; Hochgrafe et al., 2013).

From a therapeutic point of view, besides manipulating TAU levels, its localization and the interaction with other proteins, a range of alternative strategies can be pursued. One is restoring neuronal functions by transplanting stem cells to provide neurotropic support (Yamasaki et al., 2007). As far as TAU is concerned, evidence is accumulating that its phosphorylation is critical for TAU to be toxic, and that reducing TAU phosphorylation is a promising strategy (Gong and Iqbal, 2008). What is not entirely clear is whether this requires distinct phosphorylation events or whether a generally elevated level of phosphorylation is sufficient. The latter possibility is suggested by work in Drosophila (Steinhilb et al., 2007). In principal, one would either like to inhibit TAU kinases such as GSK3 (Engel et al., 2006), or activate TAU phosphatases such as PP2A (Delobel et al., 2002), or both. In fact, the PP2A activator sodium selenate that dramatically reduced TAU pathology in several mouse models (van Eersel et al., 2010) is currently evaluated in a phase IIa clinical trial in mild to moderate AD (https://www.anzctr.org.au/Trial/Registration/TrialReview.aspx?id=83952).

Other suitable therapeutic strategies are the restoration of mitochondrial function, blocking the interaction of TAU with FYN or JIP1, or disrupting the excitotoxic complex of NMDAR and PSD-95. Other strategies are the activation of chaperones (Ward et al., 2012) and the use of aggregation blockers (Akoury et al., 2013). While it is clear that Aβ plays a key role in the pathogenesis of AD, it is becoming even clearer that there is a future of therapeutics for AD beyond amyloid (Lane et al., 2012; Yoshiyama et al., 2012). Whether in practical terms it will be necessary for the efficacy of emerging therapeutic strategies to exactly determine which TAU species is the most toxic form, remains to be determined.

## Conflict of Interest Statement

The authors declare that the research was conducted in the absence of any commercial or financial relationships that could be construed as a potential conflict of interest.
